# Moxas of Marmoral (M. Guèpratte)

**Published:** 1844-10-19

**Authors:** 


Moxas of Marmoral (M. Guèpratte).—Their preparation is simple, their application convenient, and their action can be regulated at the will of the operator. A leaf of ungummed paper, soaked in subacetate of lead, and properly dried, is sufficent to prepare sixty cylinders, which will burn alone always parallel to their bases, and with sufficient slowness to develope gradually that heat, which ought to crack the epidermis and produce an eschar. There are neither sparks nor smoke to annoy the practitioner, nor to inconvenience the patient, and bring on the fits of coughing of phthisical patients, for instance. M. Guèpratte generally substitutes new unbleached calico for the paper; he cuts slips about half as high again as the moxas; he folds, and rolls them round, securing the last fold with a few stitches. In this manner he has not a perfect cylinder, but a truncated cone, with a sufficiently large base not to require the use of any instrument to retain its position, which it can do itself if the skin be moistened. All that is requisite is to hold the patient steady.—*Ibid.*

